# Plastispheres as reservoirs of antimicrobial resistance: Insights from metagenomic analyses across aquatic environments

**DOI:** 10.1371/journal.pone.0330754

**Published:** 2025-09-03

**Authors:** Ingun Lund Witsø, Amulya Baral, Ann-Katrin Llarena, Marina Aspholm, Mette Myrmel, Yngvild Wasteson

**Affiliations:** 1 Faculty of Veterinary Medicine, Department of Paraclinical Sciences, Norwegian University of Life Sciences, Ås, Norway; 2 Faculty of Veterinary Medicine, Department of Production Animal Clinical Sciences, Norwegian University of Life Sciences, Ås, Norway; CSSRI: Central Soil Salinity Research Institute, INDIA

## Abstract

Evidence suggests that plastic particles from various environments can accumulate harmful microorganisms and carry bacteria with antimicrobial resistance genes (ARGs). The so-called “plastisphere” might facilitate the spread of pathogens and antimicrobial resistance across environments, posing risks to human and animal health. This study aimed to analyze the diversity and abundance of ARGs found in plastispheres from various aquatic environments, identify clinically relevant pathogenic species, and ascertain bacterial hosts carrying ARGs. We present data from 36 metagenomes collected from plastispheres in different environments (freshwater, raw wastewater, and treated wastewater). The diversity and abundance of ARGs in the resistome of the plastispheres were analyzed through metagenomic methods. A total of 537 high-quality metagenomic-assembled genomes (MAGs) were constructed to identify clinically relevant pathogens and to link the detected ARGs to their bacterial hosts. The results show that the environment has the greatest influence on the abundance and diversity of ARGs in the plastispheres resistome, with the wastewater plastisphere containing a resistome with the highest diversity of ARGs. Resistance to beta-lactams, aminoglycosides, and tetracyclines were the most abundant resistance mechanisms detected in the different plastispheres. The construction of MAGs identified potential pathogens and environmental bacteria that confer resistance to one or several drug classes, with beta-lactams being the most pervasive form of AMR detected. This work enhances our understanding of the plastisphere’s role in antimicrobial resistance dissemination and its ecological and public health risks.

## Introduction

Plastic production has increased in recent decades, leading to subsequent environmental pollution with plastic debris, which is currently one of the planet’s ecosystems’ challenges. Since 2015, approximately 6,300 million metric tons of plastic have been produced, with 79% discarded and accumulated in natural environments and landfills [1,2]. Plastic is considered an accumulating and poorly reversible pollutant, and the pollution affects various habitats, including aquatic systems. The weathering of plastic starts immediately as it enters the environment and depends on the properties of the plastic and the environmental conditions. Despite several strategies implemented to reduce plastic pollution, plastic enters the environment through several routes, such as inadequate plastic waste handling and ineffective plastic retention through wastewater treatment [3]. Municipal wastewater treatment plants (WWTPs) receive plastic particles of varied sizes from residential and industrial activities. Although filtering, coagulation, and sedimentation will remove large and small plastic fragments from the influent wastewater, no treatment achieves complete plastic retention [4]. Consequently, wastewater effluent introduces plastic contamination into aquatic environments such as fjords, oceans, and rivers [5–7]. Plastic particles entering aquatic environments can be ingested by vertebrates and dispersed through the food chain, potentially having adverse effects on the ecosystem [8,9]. Additionally, treated wastewater used for irrigation in water-scarce regions can introduce plastics into the food chain, contaminating crops and fresh produce consumed by humans [10,11].

Plastics in the environment attract and accumulate inorganic and organic substances on their surfaces, forming a diverse microbial community known as plastispheres [12]. These biofilms create an “eco-corona”; a layer of molecules and microorganisms that covers the plastic surface [13]. The surface chemistry of plastics affects the microbial composition and behavior [12,14], offering advantages such as enhanced nutrient access, protection from desiccation, and increased opportunities for gene exchange [15]. However, plastispheres also harbor potentially pathogenic bacteria such as *Escherichia coli*, *Salmonella enterica, Klebsiella pneumoniae, Listeria monocytogenes*, and *Acinetobacter* spp. [16–18].

The emergence of antimicrobial resistance (AMR) in diverse bacterial populations poses a severe threat to the effective treatment of infectious diseases, described as a “silent pandemic” and a pressing global health crisis [19]. Addressing AMR requires multifaceted approaches and should be studied alongside the plastic pollution challenge. Plastispheres can act as reservoirs and vectors for antimicrobial resistance genes (ARGs), facilitating their accumulation and dispersal across environments [20,21]. Various ARGs have been identified in plastic biofilms [22,23], including those conferring resistance to vancomycin, sulfonamides, and beta-lactams [22–24]. Thus, the association between plastispheres and ARGs becomes increasingly apparent. In addition to the physical presence of plastic contamination, which has raised concerns among environmental scientists and public health experts [25], the plastisphere’s potential role as vector for ARGs presents an alarming connection between plastic pollution and the global AMR health crisis [26].

This study addresses critical knowledge gaps related to the plastisphere’s resistome and its accumulation in different aquatic environments. Although metagenomic sequencing has increasingly become the preferred technology when characterizing microbiomes and resistomes [22,26,27], previous investigations into the plastispheres’ role as a vehicle for pathogens and AMR have largely employed amplicon sequencing for taxonomic characterization [28], alongside qPCR and phenotypic assays to assess microbial pathogenicity and resistance profiles [29]. While these methods provide valuable insights, they offer limited resolution into the full microbial community structure and are insufficient to precisely link the ARGs to a specific bacterial host.

To address these limitations, this study employed a comprehensive metagenomic approach to investigate the resistome of plastispheres from different aquatic environments, including freshwater, raw wastewater, and treated wastewater.

The specific aims were to: 1) Analyze the diversity of the resistome found in plastispheres from various aquatic environments, 2) Examine variations in the abundance of ARGs in plastispheres, 3) Identify clinically relevant pathogenic species, identified as the ESKAPEE pathogens within the plastispheres; and 4) Use an assembly-based approach to identify bacterial hosts carrying ARGs. Altogether, this study provides a deeper understanding of the plastisphere’s potential role in the dissemination of AMR, offering critical insights into its ecological and public health implications.

## Materials and methods

### Plastisphere sampling and DNA purification

The plastisphere resistomes analyzed and characterized in this study are from DNA samples previously subjected to 16S amplicon sequencing in previous studies [17,18], where the taxonomic diversity and composition of the plastisphere were described.

Below is a brief description of the protocols, while more detailed descriptions of the sampling procedure and DNA extraction can be found in the Supporting Information (S1 and S2 Files).

Freshwater, raw, and treated wastewater were used as environments to study the resistome of the plastispheres. Biofilm accumulated on plastic pieces placed into these environments for two and four weeks (S1 Fig). The duration of incubation was based on a pilot study, where the amount of biofilm formed was insufficient for the extraction of sufficiently high-quality DNA, after a shorter time. At harvesting, the pieces were collected in sterile containers filled with river water for the river samples and PBS for the wastewater samples and transported to the laboratory within two hours. The pieces were rinsed carefully three times with distilled water to remove loosely attached organic material and frozen at −80°C for later extraction of DNA. Freshwater samples (Lier River) were labelled as R (for “river”) followed by a number (Table A in S2 File). Plastisphere samples from VEAS (treatment plant) were labelled as V (for “VEAS”) followed by a number, distinguishing the samples from the different environments. The raw wastewater is referred to as “raw WW”, and the effluent wastewater is referred to as “treated WW” throughout the text. The samples from VEAS were obtained after access was approved by the VEAS treatment plant. The sampling sites in the Lier River were located at public recreational sites, requiring no permits.

### Shotgun metagenomic sequencing and data processing

A commercial sequencing company performed sample quality control and shotgun metagenomic sequencing of extracted DNA (Novogene, Cambridge, United Kingdom). The genomic DNA was randomly sheared into short fragments, and further end-repaired, A-tailed, and ligated with Illumina adapters. The fragments with adapters were size-selected, PCR amplified, and purified. The library was quantified using Qubit and qPCR, and size distribution was determined using Bioanalyzer. The libraries were further pooled and sequenced using a NovoSeq 6000 Illumina platform with a paired-end (2 × 150 bp) strategy, according to the effective library concentration and data amount required.

### Bioinformatics and quality control (QC)

All bioinformatics analyses with in-house scripts and pipelines were performed at the Orion High-Performance Computing (OHPC) Cluster at the Norwegian University of Life Sciences (NMBU), and general data manipulation was done in R (v4.4.0) and RStudio (v2024.04). A flow chart of the bioinformatics pipeline is shown in S2 Fig.

Paired-end quality-and-adapter-trimmed sequences (150 bp) were obtained from the sequencing company. To ensure the reads met the required quality standards, an additional round of QC was performed with FastQC v0.12.1. Bowtie2 (v2.4.1) was used to check for potential PhiX as well as human contamination by mapping reads to the human genome (GCA_000001405.15_ GRCh38) [30]. No PhiX contamination was detected.Only reads that did not map to the human genome were kept for further analysis using the --un-conc parameter.

### Detection and quantification of antimicrobial resistance genes from reads

Reads were aligned to the Nucleotide_Fasta_Protein_Homolog_Model of the Comprehensive Antibiotic Resistance Database (CARD) v3.2.6 using KMA v1.4.9 [31] with the following arguments: kma -ipe -mem_mode -ef -1t1 -cge -nf –vcf. KMA uses a k-mer-based alignment approach with its ConClave scoring algorithm, which makes it effective for aligning ARGs against redundant databases like CARD, where multiple homologous resistance genes must be accurately distinguished. To focus on high-confidence hits, the AMR hits were filtered to include only those with template identity and coverage ≥ 80%. ARG read counts per sample were adjusted according to their sample-specific number of Bracken reads assigned to the super Kingdom Bacteria. The adjusted reads were used to calculate the relative abundance as Reads Per Kilobase of reference gene per Million bacterial reads (RPKM) [32]:


$$RPKM\;=\;\frac{ARG\;read\;count\;}{ARG\;length\;\times \;Bacterial\;read\;count\;}\times \hat{10}\;9$$

### Taxonomic classification of reads

Taxonomic classification was performed using Kraken2 v2.1.3 [33] with the standard database (k2_standard_20231009). Bracken (v2.9) was used to refine Kraken2 read counts by re-estimating species abundance based on the k-mer distribution [34].

The “potentially pathogenic bacteria” were defined as the species that include clinically relevant pathogens *Enterococcus faecium, Staphylococcus aureus, K. pneumoniae, Acinetobacter baumannii, Pseudomonas aeruginosa, Enterobacter* spp. and *E. coli* (referred to as the ESKAPEE pathogens) [35–37].

### Taxonomic context of the ARGs and metagenome assembled genomes (MAGs)

*De novo* assembly of the metagenomic reads was performed using MEGAHIT (v1.2.9) [38] with filtering of contigs shorter than 1000 bp. The quality of the assembled contigs was evaluated using the metaquast module within QUAST (v5.2.0) [39] before downstream processing.

Contigs were aligned against the CARD (v3.2.6) using BLAST+ (v2.15.0) [40]. Contigs with hits were retained for further analysis when the percentage identity and coverage (template length/subject length) were ≥80%. This cutoff was selected to optimize the balance between sensitivity (detecting known ARGs with minor sequence variations or incomplete read coverage) and specificity (minimizing false positives from short, non-specific alignments), a commonly used criterion [41,42]. Contigs that contained ARGs surrounded by flanking sequences ≥1500 bp on either side of the ARG were extracted using an in-house Linux bash script (available at GitHub - amulyabaral/plastpath: scripts and graphs for plastpath metagenome analysis). All sequence contigs with ARG hits, except for one, passed this requirement and were subsequently classified using Kraken2 with the standard Kraken database as described above.

The assembled contigs were binned into MAGs using MetaBat2 (v2.15, Bioconda) [43] with parameters: minimum contig length of 1500 bp, minimum contig variance 1.0, minimum contig variance sum 1.0, maximum probability 95%, minimum score 60, maximum edges 200, and minimum cluster size 200,000. Coverage information for MetaBat2 was calculated using jgi_summarize_bam_contig_depths function within MetaBat2 from BAM files generated by read-mapping the contigs with Bowtie2 (v2.4.1) and SAMtools (v1.20) [44]. The quality and completeness of the resulting MAGs were assessed using CheckM (v1.2.2) [45] lineage workflow with default parameters. MAGs were considered high quality if they had ≥ 80% completion and ≤10% contamination. Only high-quality MAGs were selected for downstream analysis. Taxonomic classification of the MAGs was performed using GTDB-Tk (v2.3.2) The assembled contigs were binned into MAGs using MetaBat2 (v2.15, Bioconda) [43] with the following parameters: minimum contig length of 1500 bp, minimum contig variance 1.0, minimum contig variance sum 1.0, maximum probability 95%, minimum score 60, maximum edges 200, and minimum cluster size 200,000. Coverage information for MetaBat2 was calculated using jgi_summarize_bam_contig_depths function within MetaBat2 from BAM files generated by read-mapping the contigs with Bowtie2 (v2.4.1) and SAMtools (v1.20) [44]. The quality and completeness of the resulting MAGs were assessed using CheckM (v1.2.2) [45] lineage workflow with default parameters. MAGs were considered high quality if they had ≥ 80% completion and ≤10% contamination. Only high-quality MAGs were selected for downstream analysis. Taxonomic classification of the MAGs was performed using GTDB-Tk (v2.3.2) [46] with the GTDB release 214 reference database. ARGs in the MAGs were identified using BLASTN against the CARD database. Hits were retained when the percentage identity and coverage (template length/subject length) was > 80%. To avoid redundant hits, the results were deduplicated by retaining only the highest-scoring hit for each 100 bp region of the query sequence.

### Statistical analysis and data visualization

The statistical analyses were performed using RStudio software v4.3.0. [47].

Based on the previous studies using 16S sequencing, where no statistically significant effect of plastic material was found on the plastispheres [17,18], we combined the samples from the different plastics and considered them as “plastic”. Thus, we had three replicates for each variable (S1 Table). The Shannon and Simpson diversities of the plastisphere resistome were calculated and visualized using *vegan* and *tidyverse* packages [48,49]. Significant differences in the alpha diversities of the resistomes were evaluated using non-parametric tests, as the data did not meet the assumption of normality (Shapiro-Wilk test, *p* < 0.05). Therefore, a rank transformation was applied to the data, followed by a parametric analysis of variance (ANOVA) model on the ranks. The alpha measures were used as response variables, while the associated variables “Environment” and “Duration” were used as explanatory variables. Tukey HSD post hoc tests were performed to conduct multiple comparisons on rank-transformed data. Ties in the data were handled by assigning average ranks using the rank () function in RStudio [48]. Statistical significance was set at *p* < 0.05. The results were plotted using the package *ggplot2* in RStudio [50].

Nonparametric permutational multivariate analysis of variance (PERMANOVA) based on a Bray-Curtis dissimilarity matrix with 999 permutations was performed to test statistical differences between the resistomes in the plastispheres. The analysis was done using the *adonis2* function (*vegan* package) in RStudio. To test whether differences in the ARGs composition were influenced by within-group variability dispersion, a permutational analysis of multivariate dispersion (PERMDISP) test was used. The test was performed using the function “*betadisper*”. A Tukey post hoc test was conducted for pairwise comparison of group dispersions to identify which groups exhibited significant differences using the “Tukey HSD” function. Adjusted p-values were reported to control for multiple testing. Tukey HSD calculates the adjusted p-value using the Studentized Range Statistic. The resistome composition was visualized using a non-metric multidimensional scaling (NMDS) plot based on Bray–Curtis dissimilarity.

Statistical analysis of the ESKAPEE pathogens was performed using a non-parametric Kruskal-Wallis test, with “Environment” as the associated variable. The Tukey HSD post hoc test was used for pairwise comparison of the environments.

## Results

Pieces of three different plastic materials were submerged in freshwater, raw WW, and treated WW environments. Plastic-associated biofilms were then collected after incubation periods of two and four weeks. DNA was extracted from the plastispheres, followed by metagenomic sequencing. 36 metagenomes were generated. The sequencing effort yielded an average of 70.1 million reads per sample (SD = 8,551,595), with read counts ranging from 47.3 million to 85 million reads. Detailed information about per-sample read counts and quality are available in the supplementary data (S2 Table).

### The resistome of river water and wastewater plastispheres

#### Diversity of the resistome in the plastispheres.

**Fig 1:**
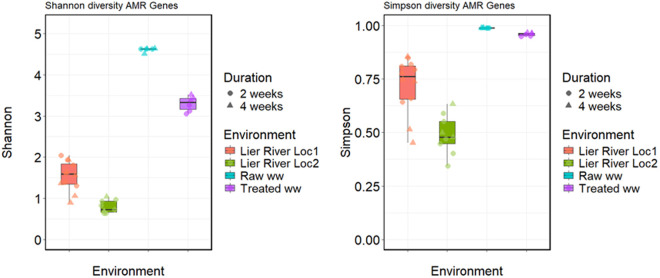
The alpha diversity estimates of the resistance gene identified in the plastispheres from river water and wastewater. Boxplots show the distribution of **(A)** Shannon diversity (richness and evenness) and **(B)** Simpson diversity (the abundance) values across different environments. The color indicates the different environments from which the plastispheres were collected, with individual samples represented as jittered points. The point shape represents the duration of incubation. The boxes show the interquartile range, with the horizontal line representing the median value.

Two ANOVA models were performed with “Environment” and “Duration” as explanatory variables and the two alpha diversity indices as response variables. The results from both models indicated that the environment had the greatest influence on alpha diversity (Fig 1). The ARGs in the wastewater plastispheres were more diverse (Shannon and Simpson diversity) than those in freshwater plastispheres (Tables B and C in S2 File). The Shannon diversity of the ARGs was higher in the plastispheres from raw WW compared to treated WW and river water, suggesting the presence of rare ARGs in raw WW plastispheres. The similar Simpson diversity of ARGs from raw and treated WW plastispheres reflects a more balanced abundance between dominant and common ARGs. In contrast, the lower alpha diversity in freshwater plastispheres suggests a reduced number of ARGs, with a few ARGs dominating.

**Fig 2:**
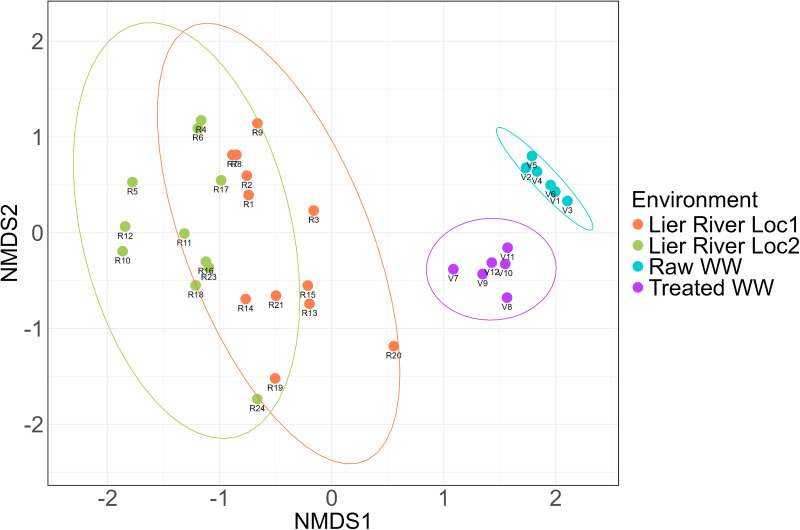
Composition differences of the antimicrobial resistance genes in the plastispheres. A non-metric multidimensional scaling (NMDS) plot illustrates the Bray-Curtis distances of the antimicrobial resistance genes composition in the plastispheres across the environments. The samples are shown with points and ellipses colored by the environment. A statistically significant PERMANOVA result (*p* < 0.001) indicates distinct ARG composition between the environments. The spread of points within each environment reflects variability in the composition of ARGs, supported by a statistically significant PERMDISP result (*p* = 7.354e-07).

Bray-Curtis dissimilarity distance was applied in PERMANOVA to assess beta-diversity differences in the ARG profile of the plastispheres. The initial model included two explanatory variables (“Environment” and “Duration”) and their interaction. The environment explained over 50% of the variation of the ARG in the plastispheres (Table D in S2 File, *p* < 0.001), while no significant interaction effect was observed between the variables. Dispersion analysis revealed significant within-group variability in ARG composition across environments, but the variability was not affected by the duration of the plastic pieces spent in each environment (Table E in S2 File). The ARG profiles from the plastispheres in raw and treated WW differed significantly from those in river water. These results are visualized in the NMDS plot (Fig 2). The resistomes cluster according to the environment, supporting the significant PERMANOVA results (Fig 2, Table D in S2 File). However, the plot also shows that the variation of the ARGs in the resistome from freshwater and treated WW is larger than the raw WW resistome, consistent with the PERMDISP results (*p* < 0.001, Tables E and F in S2 File).

An additional PERMANOVA was conducted to investigate the variation within each environment separately. The plastispheres from the river water were collected during spring (June) and fall (September); thus, the variable “Month” was included as an explanatory variable in the model. The results show that the location (“Environment”) in the river and the month when the plastispheres were collected had a significant effect on the variation of the ARGs (Table G in S2 File). The variation of the ARGs in the plastispheres from wastewater was further investigated, with “Environment” and “Duration” as explanatory variables. The environment was the only variable that had a statistically significant impact on the diversity of the ARGs in the WW plastispheres (Table H in S2 File).

#### The abundance of ARGs in wastewater and riverine plastispheres.

The results from the diversity analysis revealed a statistically significant impact of the environment on the ARG diversity in the plastispheres; therefore, we chose to focus on the differences between the environments regarding the abundance of ARGs.

**Fig 3:**
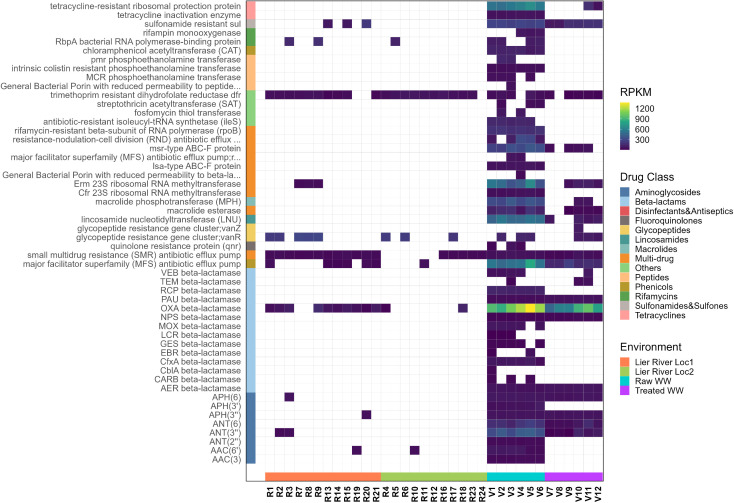
Relative abundance of ARGs.

Overall, 191 ARGs from 21 different drug classes conferring resistance through six distinct mechanisms were identified (Fig 3, S3 Fig, Table I in S2 File). The beta-lactams were the most abundant drug class in the WW plastispheres, followed by aminoglycoside, tetracycline, macrolides, and lincosamide. These results coincide with previous reports on the composition of the wastewater resistome [26,51]. For the freshwater plastispheres, the glycopeptides were the most abundant ARG drug class (Fig 3, S3 Fig, Table J in S2 File).

The heatmap shows the relative abundance of ARGs across different samples. The blue-to-yellow color scale represents the abundance of reads measured in reads per kilobase per million (RPKM). On the left side of the heatmap, a colored column indicates the drug classes to which the genes confer resistance. The antimicrobial resistance gene families are listed on the y-axis. The samples are shown on the x-axis, with their corresponding environments represented by a colored row at the bottom of the heatmap.

**Fig 4:**
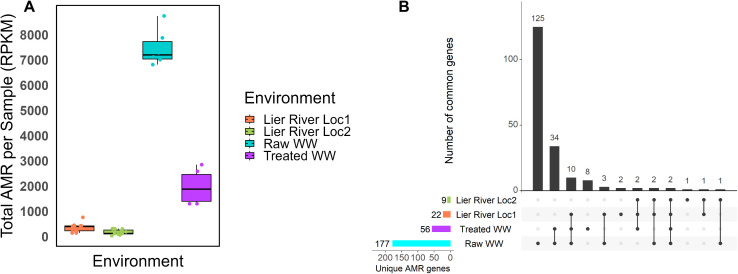
The number of ARGs found in the plastispheres. **(A)** The box plot shows the total AMR load (RPKM) across the plastispheres from different environments. The number of ARGs in raw WW (n = 177) was statistically significantly higher compared to the number of ARGs from treated WW (n = 56, p. adj = 0.005) and river water plastispheres (Loc1: n = 22, p. adj = 9.9e-4. Loc 2: n = 9, p. adj = 9.9e-4). **(B)** The UpSet plot shows the number of unique and shared antimicrobial resistance genes detected in the plastispheres across different environments.

The abundance of the different ARGs differed between the environments (Kruskal-Wallis test, *p* = 6.714e-07). Plastispheres from raw WW contained a higher number of ARGs (n = 177) compared to those from treated WW (n = 56, p. adj = 0.005) and river water (Loc1: n = 22, p. adj = 9.9e-4. Loc 2: n = 9, p. adj = 9.9e-4) (Fig 4A). Among all identified ARGs, two (1%) were found to be common across all environments (Fig 4B), namely *quaE* (deltaE) and *quaL*, belonging to the drug class “disinfectant agents and antiseptics”. Among all detected ARGs, 65% (n = 125) were unique to the plastispheres from raw WW. The most frequently occurring ARGs were associated with beta-lactams (n = 28), aminoglycosides (n = 17), lincosamides (n = 15), and tetracyclines (n = 15). Additionally, 4.2% of the ARGs detected were unique to the treated wastewater plastispheres, with most of them falling under the drug classes beta-lactams, cephalosporins, and glycopeptides. Furthermore, 17.8% (n = 34) of the ARGs were shared between the plastispheres from raw and treated wastewater.

In the plastispheres from the two river locations, two (1%) and one (0.5%) ARGs were unique to each location. All three of these ARGs were beta-lactams.

The most abundant ARGs detected in the raw WW plastisphere were *mphE* (macrolide phosphotransferase), *msrE* (macrolide efflux protein), and *rpoB*_RIF (rifampicin resistance). In the treated WW plastispheres, *OXA-10* (beta-lactamase) and *tet(C)* (tetracycline resistance, efflux pump system) were most abundant. The most abundant ARGs for the river water plastispheres were *vanR* (vancomycin (glycopeptide) resistance), *dfrB* (trimethoprim resistance), and OXA-genes (beta-lactamases) (Fig 3, S4 Fig).

#### ESKAPEE pathogens in the plastispheres.

**Fig 5:**
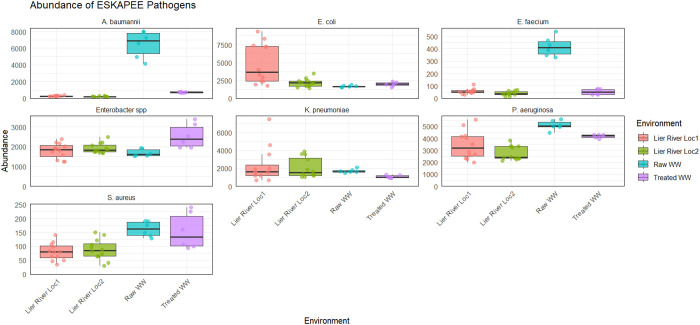
The relative abundance of ESKAPEE pathogens. The boxplots illustrate the relative abundance (calculated from RPM = reads per million) of ESKAPEE pathogens for the different environments. The line within the boxes represents the median value. The upper and lower edges of the boxes represent the higher and lower quartiles, respectively. The single points on the diagram show all the data points included in the analysis.

The presence of the ESKAPEE pathogens in the plastispheres was investigated due to their clinical relevance as emerging pathogens. These pathogens were identified across all environments (Fig 5). There was no difference in the relative abundance of *K. pneumoniae* across the environments (*p* = 0.05352) (Table K in S2 File). For *E. coli*, the relative abundance was highest in the plastisphere from river water (Loc1). The relative abundance of *Enterococcus faecium*, *A. baumannii,* and *P. aeruginosa* was highest in the raw WW plastispheres. The relative abundance of *S. aureus* was significantly higher in WW plastispheres (raw and treated) than in river water plastispheres. *E. faecium* and *S. aureus* had the lowest relative abundance of the ESKAPE pathogens, while *P. aeruginosa* had the highest abundance across all environments. A summary of these results is visualized in Fig 5 and Table K in S2 File.

### Metagenome-assembled genomes (MAGs) reveal the plastispheres as reservoirs for AMR

We recovered 537 high-quality metagenome-assembled genomes (MAGs) across 36 samples. Genomic statistics, taxonomic classification, and the presence of identified ARGs for each MAG can be found in supplementary data (S1 Table). Wastewater plastispheres (raw and treated) yielded roughly equal numbers of MAGs, with 161 and 159 MAGs, respectively. The Lier River locations showed varying MAG yields, 147 MAGs from Loc2 and 70 MAGs from Loc1.

Taxonomic classification of these MAGs using GTDB identified 22 bacterial phyla. Classification success decreased with taxonomic resolution, while all bins were classified at phylum level, only 17.32% achieved species-level classification. Pseudomonadota dominated the community structure at the phylum level (47.7% of the MAGs), followed by Bacteroidota (14.7%) and Actinomycetota (11.0%).

Raw wastewater exhibited the highest phylogenetic diversity among the recovered MAGs, comprising 15 phyla and 57 genera. Treated wastewater showed reduced diversity, with 11 phyla and 39 genera, while river locations demonstrated even lower diversity. Loc2 contained 9 phyla and 44 genera, whereas Loc1 harbored 7 phyla and 30 genera. Chi-square analysis confirmed significant differences in phylum distribution across environments (χ² = 310.48, df = 63, p < 2.2e-16). Notably, Cyanobacteriota was exclusively found in Lier River loc2 (3.35%), and Campylobacterota was unique to Treated WW (0.56%). While Pseudomonadota dominated across all environments, its abundance varied considerably: Treated WW (16.2%), Lier River loc2 (14.0%), Lier River loc1 (9.31%), and raw WW (8.19%).

33.5% of MAGs (180) carried at least one resistance gene. MAGs from treated WW showed the highest prevalence, with 40.3% carrying AMR genes (103 gene hits across 64 MAGs). River Loc2 displayed similar proportions (41.5%, 88 gene hits across 61 MAGs), while raw WW and River Loc1 showed lower frequencies (23.6% and 24.3%, respectively). The taxonomic distribution of AMR genes spanned 11 bacterial phyla, with Pseudomonadota being the primary carrier across environments. This phylum showed prominence in AMR carriage in River Loc2 (38 AMR gene hits) and treated WW (36 AMR gene hits). Bacteroidota carried a substantial AMR gene load in treated WW (49 gene hits) and River Loc2 (20 gene hits). Raw WW displayed the most diverse distribution of AMR-carrying bacteria across eight phyla. In contrast, treated WW showed a concentrated distribution, with Bacteroidota and Pseudomonadota harboring 82.5% of all AMR gene hits. River locations exhibited distinct patterns - Loc2 distributed AMR genes across six phyla, while Loc1 showed limited distribution with Pseudomonadota predominance (26/28 genes).

**Fig 6:**
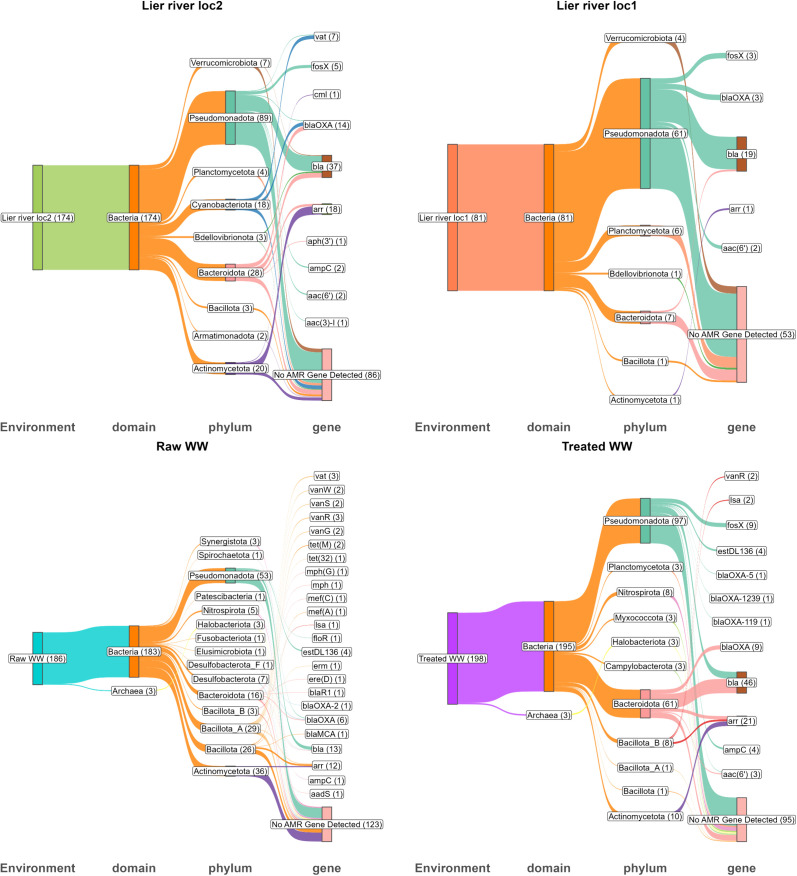
Distribution of ARGs across MAGs in different environments. The Sankey diagrams show the MAGs generated for each sampling location, their respective phyla across domains, and detected ARGs. No ARGs were detected in some MAGs, as indicated by the “No AMR Gene Detected” category. The number of MAGs corresponding to each node is shown in brackets for each node.

The treatment process appeared to select for specific AMR-carrying taxa, evidenced by the higher proportion of AMR-positive MAGs in treated versus raw WW (40.3% vs 23.6%), despite raw WW showing greater taxonomic diversity in AMR carriers. An overview of MAGs with their domain and phylum-level classification and their associated AMR genes is shown in Fig 6.

**Fig 7:**
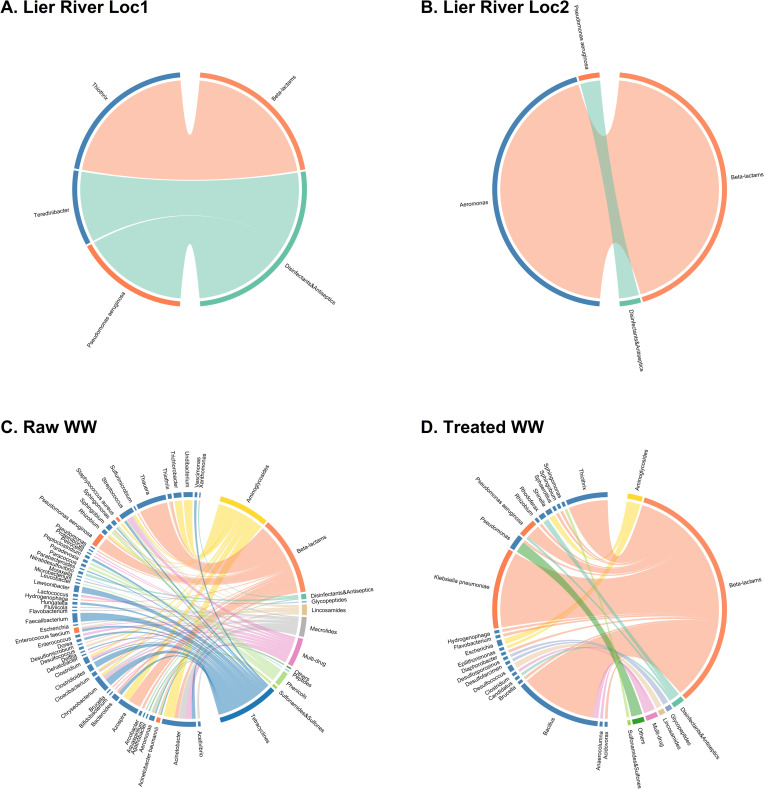
Antimicrobial resistance genes associated with bacterial hosts. Network analysis represents identified MAGs in the plastispheres from the different environments: **(A)** Lier River Loc1, **(B)** Lier River Loc2, **(C)** Raw WW, and **(D)** Treated WW. The bacteria were grouped at the genus level, while species-level analysis was maintained for the ESKAPE group and *E. coli*. The bacterial genera are listed on the left side of the network. The identified genera are marked in blue, while the ESKAPEE pathogens are highlighted in orange. The associated antibiotic resistance drug classes are listed on the right side of the network and marked with different colors.

In addition to the MAGs, assembly contigs containing both resistance genes and substantial flanking regions (>1500 bp) were analyzed to associate resistance genes with their bacterial hosts (Fig 7). While most bacteria were grouped at the genus level, species-level analysis was maintained for the ESKAPEE group. The distribution and specificity of resistance varied among the different genera. Some genera exhibited resistance to one single drug class, while others carried genes conferring resistance to multiple antibiotic types. Beta-lactam resistance emerged as the most pervasive form of AMR, occurring frequently in typical environmental bacteria and taxa associated with pathogenic bacteria (Fig 7, S3 Fig). Beta-lactam resistance genes were detected in 16 genera, followed by aminoglycoside resistance genes in 13 genera and tetracycline resistance genes in 12 genera. Macrolide resistance genes were identified in seven genera, while sulfonamide resistance genes appeared in three genera. Alarmingly, contigs from ten different genera carried multi-drug resistance genes, spanning both environmental and potentially pathogenic isolates. This underscores the widespread presence of multi-drug resistance within these environments.

In addition to the clinically relevant bacterial species carrying ARGs, multiple ARGs were detected in nonpathogenic environmental bacteria. Many environmental genera carried resistance genes across different drug classes, suggesting a broad and diverse resistance profile. For instance, *Chryseobacterium* contigs contained genes conferring resistance to aminoglycosides, macrolides, tetracyclines, and multi-drug resistance determinants. *Azospira* harbored both aminoglycoside and beta-lactam resistance genes. *Thauera* and *Thiothrix*, typically associated with WW treatment systems, carried beta-lactam resistance genes. *Undibacterium* and *Trichlorobacter* carried aminoglycoside resistance genes.

## Discussion

### Environmental conditions drive the diversity and abundance of ARGs in plastispheres

The analysis of the plastisphere’s resistome revealed significant differences in the diversity and abundance of ARGs, primarily driven by the environment in which the plastispheres were formed. Plastispheres from raw WW exhibited higher diversity and greater abundance of ARGs than those from treated WW and river water. Raw WW consists of household sewage, industrial or hospital wastewater, and urban runoff [52] and consequently contains a diverse microbiome originating from many different sources [17,18,53–55]. The levels of organic matter and nutrients are higher than in treated WW, providing more resources for bacterial growth and colonization, e.g., on plastic surfaces. Additionally, the microbial communities in fresh water and WW environments are distinct [56,57], which is reflected by the microbial communities in the plastispheres of each environment [32,58–61]. In recent studies using the same DNA samples for 16S rRNA amplicon sequencing to characterize bacterial communities in plastispheres [17,18], the environment explained most of the variation in community diversity. The microbial communities from raw wastewater plastispheres had higher alpha and beta-diversity compared to the treated wastewater plastispheres, indicating richer and more complex bacterial communities in raw wastewater [18]. These diversity shifts may have important implications for resistome composition, as the selective forces of wastewater treatment may differentially impact the persistence and dissemination of ARGs. Also, the two studies showed that the freshwater and wastewater environments provide plastispheres with a different community composition of detected genera [17,18]. Although the resistome is influenced by the environment, it is often considered independent of bacterial phylogeny due to the mobile nature of ARGs [17,18]. However, a correlation between the resistome composition and the bacterial taxonomic structure has been reported [32,58–61]. Beta diversity analysis of ARGs in these plastispheres revealed a lower dispersion in ARG profiles originating from WW compared to river water. The reduced dispersion indicates a more stable and consistent ARG composition, likely shaped by selection pressure, such as antibiotic residues and heavy metals, or microbial communities adapted to the wastewater environment. [62]. Conversely, the higher dispersion observed in river water plastispheres reflects the fluctuating environmental conditions and a more dynamic microbial ecosystem [63]. Seasonal variations were seen in river plastispheres, with ARG diversity differing between samples collected in June and September. The effect of season on the diversity of the plastisphere bacterial communities was observed in a previous study, where diversity changed significantly between June and September, with a higher diversity in June compared to September. [17]. These variations are probably due to environmental changes in the aquatic ecosystem, which shape the microbial composition and consequently influence the diversity of the ARGs. While the sample size is insufficient to confirm seasonal trends, these findings suggest temporal shifts in ARG diversity within river water plastispheres. We did not observe any effect of durability of the plastic in the river on ARG diversity. However, several earlier studies have shown that incubation duration and biofilm maturation play important roles in resistance and tolerance to antimicrobials. As biofilms develop, the extracellular matrix restricts antibiotic penetration, while bacteria inside undergo physiological changes, such as reduced growth rate, lower metabolic activity, and the emergence of persister cells, that contribute to increased antibiotic tolerance [64]. Additionally, structural and compositional changes in the biofilm during maturation may also influence the spread of resistance genes as reported in several reports including the following examples [65–67].

The limited number of replicates restricts the statistical power and generalizability of the results in the current study, making it difficult to discover broader patterns or exclude the possibility of stochastic variations. Additionally, the temporal resolution of the sampling intervals may not fully capture transient fluctuations in resistome composition, influenced by environmental factors such as rainfall, temperature, or pollution. Future studies, including higher-frequency sampling with increased replicate numbers, are needed to provide a clearer picture of ARG dynamics in plastisphere environments.

The abundance of ARGs varied significantly across environments, with plastispheres from raw WW containing substantially more ARGs than those from treated WW or river water. These differences highlight the impact of WW treatment and environmental context on ARG distribution. Beta-lactams were the most abundant drug class in the WW plastispheres, followed by aminoglycosides, tetracycline, macrolides, and lincosamide. Numerous ARGs, spanning nearly all common classes of antibiotics, have been detected in the WW influent and effluent (reviewed here [68]). The normal gut flora of humans and animals is a major source of ARGs, and antibiotic usage by humans and animals leads to the excretion of antibiotic residues into the WW system [69,70]. Consequently, WWTPs serve as critical interfaces between humans and the environment, acting as hotspots for the proliferation of antibiotic-resistant bacteria (ARB) and dispersion of ARGs, which are eventually discharged into receiving ecosystems [71,72]. Wastewater treatment plays a critical role in reducing waterborne pathogens and removing contaminants like antibiotics and plastics from effluents [71,73]. However, antibiotic removal has received less attention due to limited regulatory standards and the inefficiency of current processes [71,73]. Freshwater environments, which are also considered reservoirs and dissemination routes for ARGs [51,74], had significantly lower ARG abundance in the plastispheres than in the wastewater plastisphere. Despite these results, river resistome remains a critical public health concern, as rivers provide irrigation and production of drinking water, potentially facilitating ARG reintroduction to humans and animals.

In the present study, the primary aim was to characterize the resistome of plastispheres from river water and raw and treated wastewater, rather than to compare plastic-associated communities directly with planktonic or other surface-associated microbiomes. While several studies have demonstrated that plastispheres represent distinct microbial niches, often differing from surrounding water environments or other surfaces, a direct comparative analysis was beyond the intended scope of this research. It is important to acknowledge that aquatic environments are dynamic systems with continuous flow, leading to constant microbial flux. Our sampling period lasted up to four weeks, meaning that water samples represent a snapshot of the microbial composition at a given time rather than a long-term assessment of community dynamics.

Comparative studies that include planktonic and non-plastic surfaces are valuable to further clarify the uniqueness of plastisphere communities. Future research incorporating such comparisons will be necessary to deepen our understanding of how plastispheres contribute to microbial diversity and the dissemination of antibiotic resistance in aquatic environments. The present results highlight that environmental conditions, rather than exposure duration, dominate in shaping the resistome in plastispheres. The observed differences between WW and riverine plastispheres emphasize the interconnected impacts of microbial communities, environmental dynamics, and anthropogenic activities in driving the diversity and abundance of ARGs. Current challenges, including the rising prevalence of plastic pollution and inadequate waste management, require a revision of regulatory frameworks to improve WW treatment processes and ensure safe practices for effluent discharge.

### The presence of potential pathogens in the plastispheres

This study examined the presence of clinically significant ESKAPEE pathogens in plastispheres. These pathogens were detected across all environments (Fig 5), with higher relative abundances in plastispheres from raw WW than those from treated WW or river water, although some exceptions were observed. Notably, *E. coli* was most abundant in plastispheres from Loc1 in the Lier River. Previous research corroborates this finding, suggesting that Loc1 is more exposed to fecal contamination than Loc2 [17]. The presence of ESKAPEE pathogens in WW and freshwater environments has been reported in several studies [75,76] and might be explained by contamination via sewage spills, agricultural waste, and anthropogenic sources, including incorrectly discarded hospital waste [77,78]. However, most of the previously reported findings are based on planktonic samples. An exception is the work by Silva *et al* (2023) [79], in which MPs were sampled in a riverine environment upstream and downstream of a WWTP. They isolated *Enterobacter*, *K. pneumoniae,* and *E. coli* with virulence-related and AMR phenotypes from the plastic-associated biofilms, in addition to *Pseudomonas* and *Acinetobacter* [79]. Viable isolates of *E. coli*, *K. pneumoniae,* and *A. baumannii* have also been recovered from the same plastispheres analyzed in the present study [17,18]. Other studies that analyzed plastispheres from WW and river water using 16S rRNA sequencing detected genera containing potential pathogenic bacteria [55,80]. Although none of these studies specifically examined the pathogenic potential of the bacteria, their presence underscores that pathogenic bacteria can become incorporated into plastic-associated biofilms. These results point to the risk associated with the proliferation and spread of AMR when WW is used for irrigation purposes, highlighting the importance of proper treatment and handling of WW and sludge before disposal. However, further research is needed to clarify the transmission risk of pathogens to humans through environmental exposure pathways.

### MAG-based analysis of ARGs in environmental plastispheres

The presence of the ESKAPEE pathogens carrying ARGs in plastispheres from the different environments analyzed in the present study is concerning. Among the ESKAPEE pathogens, *E. faecium* carried resistance genes for lincosamide, tetracyclines, and multi-drug resistance, while *S. aureus* contigs contained only aminoglycoside resistance determinants. *A. baumannii* carried genes for resistance against beta-lactams, sulfonamides/sulfones, and tetracyclines. *P. aeruginosa* contigs carried genes for beta-lactam resistance and genes conferring resistance to disinfectants and antiseptics.

*K. pneumoniae* contigs exclusively carried beta-lactam resistance genes, and this species was also found in similar abundances within all environments. Given that beta-lactam resistance genes were detected in all environments and that *K. pneumoniae* is a well-known carrier of beta-lactamase-encoding genes [81], it can be speculated that the widespread presence of *K. pneumoniae* contributes to the frequent detection of beta-lactam resistance genes. In our previous studies, viable *K. pneumoniae* has been isolated from the WW plastispheres [18], and the phenotypic resistance profile was determined for several isolates [82]. The *K. pneumoniae* isolates showed phenotypic resistance against penicillin, ampicillin, tetracycline, trimethoprim/sulpha, and cephalosporin, among other antibiotics. Furthermore, the same study demonstrated that several of these isolates transferred AMR genes to a susceptible recipient within a plastic-associated biofilm [82]. Several of the beta-lactam-resistance genes harbored by *K. pneumoniae* are located on mobile genetic elements [83], facilitating the rapid spread to both *K. pneumoniae* and other bacterial species. ARGs detected in bacterial species found in plastispheres are often located on mobile genetic elements [22]. Studies have demonstrated that ARGs on mobile genetic elements are transferred at higher frequencies in plastispheres compared to the surrounding environments [21,23], highlighting the role of plastispheres in acquiring and disseminating ARGs and imposing an ecological risk. Treated WW samples yielded the highest ARG-associated MAGs among the studied environments. We hypothesize this could be a result of selective pressures in treated WW, which enrich simpler microbial communities dominated by resistant taxa. Consequently, these conditions could have made genome assembly and binning more feasible. However, while MAGs provide high-resolution data through the recovery of near-complete genomes that enable the identification of resistance mechanisms and the microbial taxa harboring them, they can be prone to issues related to completeness and contamination [84]. These challenges are particularly evident in complex and diverse samples. The possibly more complex and diverse microbial communities in raw WW and river environments are potentially more prone to yield fragmented or lower-quality MAGs.

We also obtained a relatively low rate of species-level classification among the MAGs, with only 17% achieving this taxonomic resolution. This constraint significantly impacts the ability to make fine-scale ecological inferences about ARG dissemination and host specificity. The limited species-level resolution stems from several factors, including the inherent fragmentation of short-read assemblies and the challenging nature of resolving closely related species from complex environmental samples. Future studies could address these limitations through long-read sequencing technologies to improve genome assembly contiguity and hybrid assembly approaches combining short and long reads. Continued expansion of reference databases with environmental isolates would also enhance taxonomic assignment accuracy. This constraint of MAG-based approaches may result in an incomplete representation of ARGs in these environments. Analysis of contigs containing ARGs with flanking regions of ≥1500 bp on either side captured a broader spectrum of resistance determinants and their taxonomic associations compared to MAG-based methods, as this approach is independent of genome binning quality thresholds. This strategy, therefore, complements MAG-based analyses for investigating the taxonomic distribution of antimicrobial resistance in environmental microbiomes.

Several of the resistance mechanisms detected in both ESKAPEE pathogens and the environmental genera are considered “natural resistance”. Resistance to antimicrobials evolved long before our antibiotic era and is driven by the constant competition between microorganisms. These ancient ARGs are preserved and still detected in microbiomes from anthropogenic contamination [85,86]. The introduction of pharmaceutical antimicrobial agents led to increased selection pressure, promoting mobilization and horizontal gene transfer of ARGs, driving the evolution and spread of resistance.

Biofilm formation protects microorganisms from environmental stressors compared to free-living microorganisms [87]. Thus, after being released into the environment, the plastic-associated lifestyle supports bacterial persistence and survival, presenting an indirect environmental risk associated with plastic pollution. Additionally, biofilm is considered a hot spot for horizontal gene transfer [88]. As a result, plastic particles may increase the risk of spreading AMR, and in the worst case, facilitate the dissemination of pathogenic bacteria with AMR genes. The circulation of ARGs among microbiomes in different environments, including those involving human and animal exposure, is considered one of the main challenges in the One Health approach [89]. The direct link between the plastisphere resistome and human and animal health remains unclear, highlighting the need for further investigation. This knowledge gap underscores the urgency for integrated strategies to address antimicrobial resistance, plastic pollution, and their potential combined impact on environmental and human health.

## Supporting information

S1 File Supplementary material and methods. All supporting material and methods referred to in the text are found in this file.
(DOCX)

S2 File Supplementary tables. All supporting tables (Tables A – K) referred to throughout the text are found in this file.
(DOCX)

S1 Table MAGs statistics with taxonomy and ARGs.
(XLSX)\

S2 Table Sample information with read counts and QC.
(XLSX)

S1 Fig **The custom-made device was used to collect plastispheres from the river.** The plastic pieces were mounted onto a costum-made device before being inserted into the river. To ensure the device was positioned vertically in the water column, a floating buoy and a weight were attached to the top and the bottom of the device, respectively.
(TIF)

S2 Fig Flow chart of bioinformatics pipeline.
(TIF)

S3 Fig The abundance of AMR drug classes. A barplot of the abundance of antimicrobial resistance drug classes in the resistome from the different environments. The length of the bars represents the percentage relative abundance of the drug class, and each color represents one drug class.
(TIF)

S4 Fig **The abundance of ARG.** A barplot of the abundance of antimicrobial resistance genes in the resistome from the different environments. The length of the bars represents the percentage relative abundance of the genes, and each color represents one gene.
(TIF)
