# Supplementary material for: Plastispheres as reservoirs of antimicrobial resistance: Insights from metagenomic analyses across aquatic environments
Source: PLoS One. 2025 Sep 3;20(9):e0330754. doi: 10.1371/journal.pone.0330754 (PMC12407464; doi:10.1371/journal.pone.0330754)
Supplement: S1 File — All supporting material and methods referred to in the text are found in this file. (DOCX) [file pone.0330754.s001.docx]

**Supplementary materials and methods**

**S1 Text. In situ incubation and sampling locations**

The resistome plastispheres analyzed and characterized in this study are from the same plastic-associated biofilm samples that were subjected to 16S amplicon sequencing reported in two previous studies [1, 2]. The plastispheres were collected on pieces (6 cm × 4 cm × 0.6 cm) of different plastic materials (polypropylene (PP), polyvinyl chloride (PVC), and high-density polyethylene (HDPE) (Astrup AS, Oslo, Norway). These materials were used as representative plastic materials usually found in the environment. The results from the two previous studies showed that neither the bacterial composition nor the diversity of the bacterial communities was influenced by the type of plastic material[1, 2]. Therefore, in this study, the biofilm from the three different plastic materials was considered "plastic biofilm," resulting in three independent replicates. Information about all the samples and variables is summarized in S1Table. The size of the plastic pieces was chosen to ensure a high amount of biofilm material to analyze. Each piece of plastic was submerged in the Lier River at two locations (Loc1 (59°45'03.3"N 10°17'06.3" E) and Loc2 (59°47'34.7"N 10°14'17.1" E)) in June and September 2021. The two sampling sites are found in dense agricultural areas with vegetable production. Loc2 is located downstream of wastewater treatment plants, while Loc1 is in a more densely populated area with large agricultural activity. The Lier River is currently recognized as the most plastic-polluted river in Norway [3]. The plastic pieces were sterilized with hypochlorite and mounted onto custom-made devices (S1 Figure) before being inserted into the river. An inflatable buoy was attached to one side while a weight was secured to the opposite side to keep the device positioned vertically in the river, ensuring the plastic pieces remain horizontally to the water's surface. Each device was secured to shore.

The wastewater plastispheres were collected at Veas, Norway's largest wastewater treatment plant, located near Oslo in September 2021. The plant treats 100-110 million m^3^ of municipal wastewater annually and treats sewage from 870,000 inhabitants and five major hospitals in the Oslo region. When wastewater and sewage enter Veas, solid objects are removed, and grit and organic matter are filtered out before the wastewater undergoes biological and chemical treatment. The treated effluent is then discharged into Oslo Fjord. The process takes about three to five hours [4]. The plastic pieces were mounted to a rope with a weight attached to the end to submerge them vertically in wastewater. The pieces were surface sterilized with hypochlorite before being lowered into the basins. The sampling locations were a pool with a continuous flow of raw wastewater (referred to as “raw WW”), and a basin containing effluent wastewater (referred to as “treated WW”).

**S2 Text. DNA extraction**

The plastic pieces with biofilm were thawed on ice, and the biofilms from both sides of three plastic pieces were pooled by scraping off the surface into a ZR BashingBead Lysis tube with 0.1- and 0.5-mm beads containing 750 µL of DNA/RNA Shield^TM^ provided in the extraction kit (ZymoBIOMICS DNA/RNA Minprep kit, Nordic BioSites AS, Norway). The samples were homogenized using FastPrep-24 (MP Biomedicals) at 6 m/s for 5 × 45 s, with 15 s breaks between each cycle while kept on ice. After homogenization, DNA was extracted using the ZymoBIOMICS DNA/RNA Miniprep Kit according to the manufacturer’s instructions. Samples containing ZymoBIOMICS DNAse/RNAse-free water were used as negative controls. The DNA was stored at -80°C until use.

The DNA samples used for 16S amplicon sequencing consisted of three replicates [1, 2]. In the metagenomic study, DNA from these three replicates was pooled to count as one sample. The main reason for this was to reduce the number of samples for practical and economic reasons. Information about sample ID from the two studies can be found in S1 Table.

**References**

1. Witsø, I.L., et al., *Wastewater-associated plastispheres: A hidden habitat for microbial pathogens?* PLOS ONE, 2024. **19**(11): p. e0312157.

2. Witsø, I.L., et al., *Freshwater plastispheres as a vector for foodborne bacteria and viruses.* Environmental Microbiology, 2023. **25**(12): p. 2864-2881.

3. Velle, G., et al., *Plast i norske elver*, in *NORCE LFI rapport nr 536.* 2024, NORCE Bergen. p. 49 pages.

4. VEAS, *Årsrapport (Annual report) 2021*. 2021.
