# Supplementary material for: Plastispheres as reservoirs of antimicrobial resistance: Insights from metagenomic analyses across aquatic environments
Source: PLoS One. 2025 Sep 3;20(9):e0330754. doi: 10.1371/journal.pone.0330754 (PMC12407464; doi:10.1371/journal.pone.0330754)
Supplement: S2 File — All supporting tables (Tables A – K) referred to throughout the text are found in this file. (DOCX) [file pone.0330754.s002.docx]

**Table A.** Sample information. The variables used for the plastisphere resistome analysis are environment, month, and incubation duration.

The same DNA samples have previously been used for 16S amplicon sequencing in two previous studies (samples R1- R24 [1], and samples V1- V12 [2]). For clarification, the old sample IDs are included to allow cross-referencing of the results.

| Sample ID | Environment | Month | Duration of incubation | Sample ID previous studies[1, 2] |
| --- | --- | --- | --- | --- |
| R1 | Lier River Loc1 | June | 2 weeks | A1 |
| R2 | Lier River Loc1 | June | 2 weeks | B1 |
| R3 | Lier River Loc1 | June | 2 weeks | C1 |
| R4 | Lier River Loc2 | June | 2 weeks | A2 |
| R5 | Lier River Loc2 | June | 2 weeks | B2 |
| R6 | Lier River Loc2 | June | 2 weeks | C2 |
| R7 | Lier River Loc1 | June | 4 weeks | D1 |
| R8 | Lier River Loc1 | June | 4 weeks | E1 |
| R9 | Lier River Loc1 | June | 4 weeks | F1 |
| R10 | Lier River Loc2 | June | 4 weeks | D2 |
| R11 | Lier River Loc2 | June | 4 weeks | E2 |
| R12 | Lier River Loc2 | June | 4 weeks | F2 |
| R13 | Lier River Loc1 | September | 2 weeks | G1 |
| R14 | Lier River Loc1 | September | 2 weeks | H1 |
| R15 | Lier River Loc1 | September | 2 weeks | I1 |
| R16 | Lier River Loc2 | September | 2 weeks | G2 |
| R17 | Lier River Loc2 | September | 2 weeks | H2 |
| R18 | Lier River Loc2 | September | 2 weeks | I2 |
| R19 | Lier River Loc1 | September | 4 weeks | J1 |
| R20 | Lier River Loc1 | September | 4 weeks | K1 |
| R21 | Lier River Loc1 | September | 4 weeks | L1 |
| R22 | Lier River Loc2 | September | 4 weeks | J2 |
| R23 | Lier River Loc2 | September | 4 weeks | K2 |
| R24 | Lier River Loc2 | September | 4 weeks | L2 |
| V1 | Raw WW | September | 2 weeks | A2 |
| V2 | Raw WW | September | 2 weeks | B2 |
| V3 | Raw WW | September | 2 weeks | C2 |
| V4 | Raw WW | September | 4 weeks | A4 |
| V5 | Raw WW | September | 4 weeks | B4 |
| V6 | Raw WW | September | 4 weeks | C4 |
| V7 | Treated WW | September | 2 weeks | D2 |
| V8 | Treated WW | September | 2 weeks | E2 |
| V9 | Treated WW | September | 2 weeks | F2 |
| V10 | Treated WW | September | 4 weeks | D4 |
| V11 | Treated WW | September | 4 weeks | E4 |
| V12 | Treated WW | September | 4 weeks | F4 |


**Table B.** Analysis of variance (ANOVA) model on the ranks. The alpha measures (Shannon and Simpson indices) were used as response variables, and the associated variables were used as explanatory variables. Statistically significant interactions are marked with bold numbers.

| Shannon | Df | Sum Sq | Mean Sq | F value | Pr(>F) |
| --- | --- | --- | --- | --- | --- |
| Environment | 3 | 2692.75 | 897.58 | 84.204 | **4.0033e-14** |
| Duration | 1 | 0.78 | 0.78 | 0.073 | 0.788 |
| Residuals | 28 | 298.47 | 10.66 |  |  |

| Simpson | Df | Sum Sq | Mean Sq | F value | Pr(>F) |
| --- | --- | --- | --- | --- | --- |
| Environment | 3 | 2580.75 | 860.25 | 58.933 | **3.227e-12** |
| Duration | 1 | 2.53 | 2.53 | 0.173 | 0.68 |
| Residuals | 28 | 408.72 | 14.60 |  |  |

**Table C.** Results from the paired post hoc Tukey test. The statistically significant interactions after ANOVA models on the ranks were analyzed with a Tukey post hoc test to assess the alpha diversity among the plastispheres. Statistically significant results are marked with bold numbers.

| Shannon | diff | lwr | upr | p adj |
| --- | --- | --- | --- | --- |
| Lier River Loc2-Lier River Loc1 | -9.916 | -13.78 | -6.053 | **1.2e-06** |
| Treated WW-Lier River Loc1 | 9.25 | 4.869 | 13.631 | **2.65e-05** |
| Raw WW-Lier River Loc1 | 15.25 | 10.869 | 19.631 | **6.2e-15** |
| Treated WW-Lier River Loc2 | 19.167 | 14.548 | 23.784 | **1.8e-16** |
| Raw WW-Lier River Loc2 | 25.166 | 20.548 | 29.784 | **3.31e-15** |
| Raw WW-Treated WW | -6.000 | -11.058 | 0.941 | **0.015** |

| Simpson | diff | lwr | upr | p adj |
| --- | --- | --- | --- | --- |
| Lier River Loc2-Lier River Loc1 | -8.75 | -13.349 | -4.150 | **9.2e-05** |
| Treated WW-Lier River Loc1 | 9.75 | 4.534 | 14.965 | **1.17e-04** |
| Raw WW-Lier River Loc1 | 15.75 | 10.534 | 20.965 | **6.2e-16** |
| Treated WW-Lier River Loc2 | 18.50 | 13.002 | 23.998 | **1.8e-13** |
| Raw WW-Lier River Loc2 | 24.50 | 19.002 | 29.998 | **3.31e-55** |
| Raw WW-Treated WW | -6.00 | -12.022 | 0.022 | 0.051 |

**Table D.** A PERMANOVA using Bray–Curtis distance was calculated to analyze the main effect of the different variables on the beta diversity of the ARG composition in the plastispheres. Statistically significant interactions are marked with bold numbers.

|  | Df | Sum Of Sqs | R^2^ | F | Pr(>F) |
| --- | --- | --- | --- | --- | --- |
| Environment | 3 | 6.1610 | 0.53849 | 12.1073 | **0.001** |
| Duration | 2 | 0.1503 | 0.01314 | 0.8862 | 0475 |
| Environment:Duration | 3 | 0.5501 | 0.04808 | 1.0810 | 3.373 |
| Residual | 27 | 4.5798 | 0.40029 |  |  |
| Total | 34 | 11.4412 | 1.000 |  |  |

**Table E.** A PERMANOVA analysis of the plastisphere from river water. The Bray–Curtis distance was calculated to analyze the main effect of the different variables on the beta diversity of the ARG composition in the plastispheres from river water. Statistically significant interactions are marked with bold numbers.

|  | Df | Sum Of Sqs | R^2^ | F | Pr(>F) |
| --- | --- | --- | --- | --- | --- |
| Environment | 1 | 0.853 | 0.148 | 4.692 | **0.006** |
| Month | 1 | 1.266 | 0.219 | 6.964 | **0.001** |
| Duration | 1 | 0.191 | 0.033 | 1.048 | 0.391 |
| Residual | 17 | 3.454 | 0.599 |  |  |
| Total | 22 | 5.765 | 1.000 |  |  |

**Table F.** A PERMANOVA analysis of the plastisphere from wastewater. The Bray–Curtis distance was calculated to analyze the main effect of the different variables on the beta diversity of the ARG composition in the wastewater plastispheres. Statistically significant interactions are marked with bold numbers.

|  | Df | Sum Of Sqs | R^2^ | F | Pr(>F) |
| --- | --- | --- | --- | --- | --- |
| Environment | 1 | 1.265 | 0.774 | 43.0972 | **0.001** |
| Duration | 1 | 0.104 | 0.063 | 3.5439 | 0.086 |
| Residual | 9 | 0.264 | 0.161 |  |  |
| Total | 11 | 1.633 | 1.000 |  |  |

**Table G.** Result from pairwise PERMDISP analysis of the variables. Statistically significant interactions are marked with bold numbers.

| Variable |  | Df | Sum Sq | Mean Sq | F- value | Pr (>F) |
| --- | --- | --- | --- | --- | --- | --- |
| Environment | Groups | 3 | 0.633 | 0.2107 | 17.619 | **7.354 e -07** |
|  | Residual | 31 | 0.3709 | 0.012 |  |  |
| Duration | Group | 1 | 0.009 | 0.009 | 2.112 | 0.156 |
|  | Residual | 33 | 0.141 | 0.004 |  |  |

**Table H.** Results from the paired post hoc Tukey test of statistically significant interactions after PERMDISP analysis, to assess multivariate dispersion of ARGs among the plastispheres. Statistically significant results are marked with bold numbers.

|  | diff | lwr | upr | p adj |
| --- | --- | --- | --- | --- |
| Lier River Loc2-Lier River Loc1 | -0.027 | -0.152 | 0.096 | 0.927 |
| Treated WW-Lier River Loc1 | -0.251 | -0.400 | -0.103 | **3.724e-04** |
| Raw WW-Lier River Loc1 | -0.331 | -0.479 | -0.182 | **6.2e-06** |
| Treated WW-Lier River Loc2 | -0.224 | -0.374 | -0.073 | **1.812e-03** |
| Raw WW-Lier River Loc2 | -0.303 | -0.453 | -0.152 | **3.31e-05** |
| Raw WW-Treated WW | -0.079 | -0.250 | 0.092 | 0.598 |

**Table I**: The total number of antimicrobial drug classes and ARGs detected in different environments.

| Environment | Drug classes | ARGs |
| --- | --- | --- |
| Lier river loc 1 | 9 | 22 |
| Lier river loc 2 | 6 | 9 |
| Raw WW | 21 | 56 |
| Treated WW | 12 | 177 |

**Table J:** The abundance of the drug classes in the different environments. The “*nd*” denotes that the specific drug class was undetected in the environment.

| **Drug class** | **Loc 1** | **Loc 2** | **Raw wastewater** | **Treated wastewater** |
| --- | --- | --- | --- | --- |
| glycopeptides | 1329.919 | 691.733 | 1530.681 | 376.271 |
| carbapenem | 758.600 | 147.074 | 7037.941 | 4677.270 |
| disinfecting agents and antiseptics | 637.021 | 228.392 | 1032.016 | 572.602 |
| diaminopyrimidine antibiotic | 435.119 | 621.796 | 1110.667 | 126.262 |
| sulfonamide antibiotic | 382.223 | *nd* | 1651.425 | 899.027 |
| rifamycin antibiotic | 330.754 | 124.332 | 627.105 | *nd* |
| aminoglycoside antibiotic | 250.238 | 46.363 | 5450.530 | 1519.644 |
| lincosamide antibiotic | 120.245 | *nd* | 6611.231 | 684.067 |
| tetracycline antibiotic | 27.803 | *nd* | 6863.746 | 1269.690 |
| macrolide antibiotic | *nd* | *nd* | 6269.097 | 529.773 |
| phenicol antibiotic | *nd* | *nd* | 1691.405 | 368.906 |
| penam | *nd* | *nd* | 1215.031 | 412.434 |
| cephalosporin | *nd* | *nd* | 828.854 | 360.434 |
| peptide antibiotic | *nd* | *nd* | 796.103 | *nd* |
| mupirocin-like antibiotic | *nd* | *nd* | 681.934 | *nd* |
| cephamycin | *nd* | *nd* | 438.539 | *nd* |
| glycylcycline | *nd* | *nd* | 431.576 | *nd* |
| phosphonic acid antibiotic | *nd* | *nd* | 164.399 | *nd* |
| fluoroquinolone antibiotic | *nd* | *nd* | 148.514 | *nd* |
| nucleoside antibiotic | *nd* | *nd* | 124.561 | *nd* |

**Table K.** Results from the statistical analysis of the abundance of ESKAPEE pathogens.

1. The data followed a normal distribution; thus, a non-parametric test (Kruskal-Wallis) was used to test the effect of the environments on the abundance of the different species. The environment had a statistically significant impact on the abundance of all the ESKAPEE pathogens, except for *K. pneumoniae.*

2. In the case of a statistically significant Kruskal-Wallis test, a pairwise post hoc test was performed to look at differences between the different environments.

The statistically significant results are highlighted in bold numbers.

| 1- Results from the Non-parametric Kruskal-Wallis test | | | |
| --- | --- | --- | --- |
|  | Kruskal-Wallis chi-squared | df | p- value |
| *E. faceium* | 16.579 | 3 | **0.001** |
| *S.aureus* | 15.293 | 3 | **0.001** |
| *K. pneumoniae* | 7.6629 | 3 | 0.053 |
| *A. baumannii* | 29.203 | 3 | **2.03e-06** |
| *P. aeroginosa* | 19.399 | 3 | **0.0002** |
| *Enterobacter sp.* | 11.471 | 3 | **0.009** |
| *E. coli* | 16.599 | 3 | **0.001** |

| 2- Pairwise post hoc test | | | | | | |
| --- | --- | --- | --- | --- | --- | --- |
|  | ***E. faecium*** | ***S. aureus*** | ***A. baumannii*** | ***P. aeroginosa*** | ***Enterobacter sp.*** | ***E. coli*** |
| Lier River Loc2-  Lier River Loc 1 | 0.133 | 0.9626 | 0.984 | 0.127 | 0.616 | **0.016** |
| Raw WW-  Lier River Loc1 | **0.0003** | **0.0014** | **0.000** | **0.014** | 0.616 | **0.001** |
| Treated WW-  Lier River Loc1 | 0.891 | **0.004** | 0.503 | 0.122 | **0.016** | **0.019** |
| Raw WW-  Lier River Loc2 | **0.0003** | **0.004** | **0.000** | **0.001** | 0.100 | 0.080 |
| Treated WW-  Lier River Loc2 | 0.524 | **0.011** | 0.341 | **0.001** | **0.019** | 0.750 |
| Treated WW-  Raw WW | **0.004** | 0.985 | **0.000** | **0.004** | **0.013** | 0.080 |

1. Witsø, I.L., et al., *Freshwater plastispheres as a vector for foodborne bacteria and viruses.* Environmental Microbiology, 2023. **25**(12): p. 2864-2881.

2. Witsø, I.L., et al., *Wastewater-associated plastispheres: A hidden habitat for microbial pathogens?* PLOS ONE, 2024. **19**(11): p. e0312157.
