# Supplementary figures and images for: Plastispheres as reservoirs of antimicrobial resistance: Insights from metagenomic analyses across aquatic environments
Source: PLoS One. 2025 Sep 3;20(9):e0330754. doi: 10.1371/journal.pone.0330754 (PMC12407464; doi:10.1371/journal.pone.0330754)

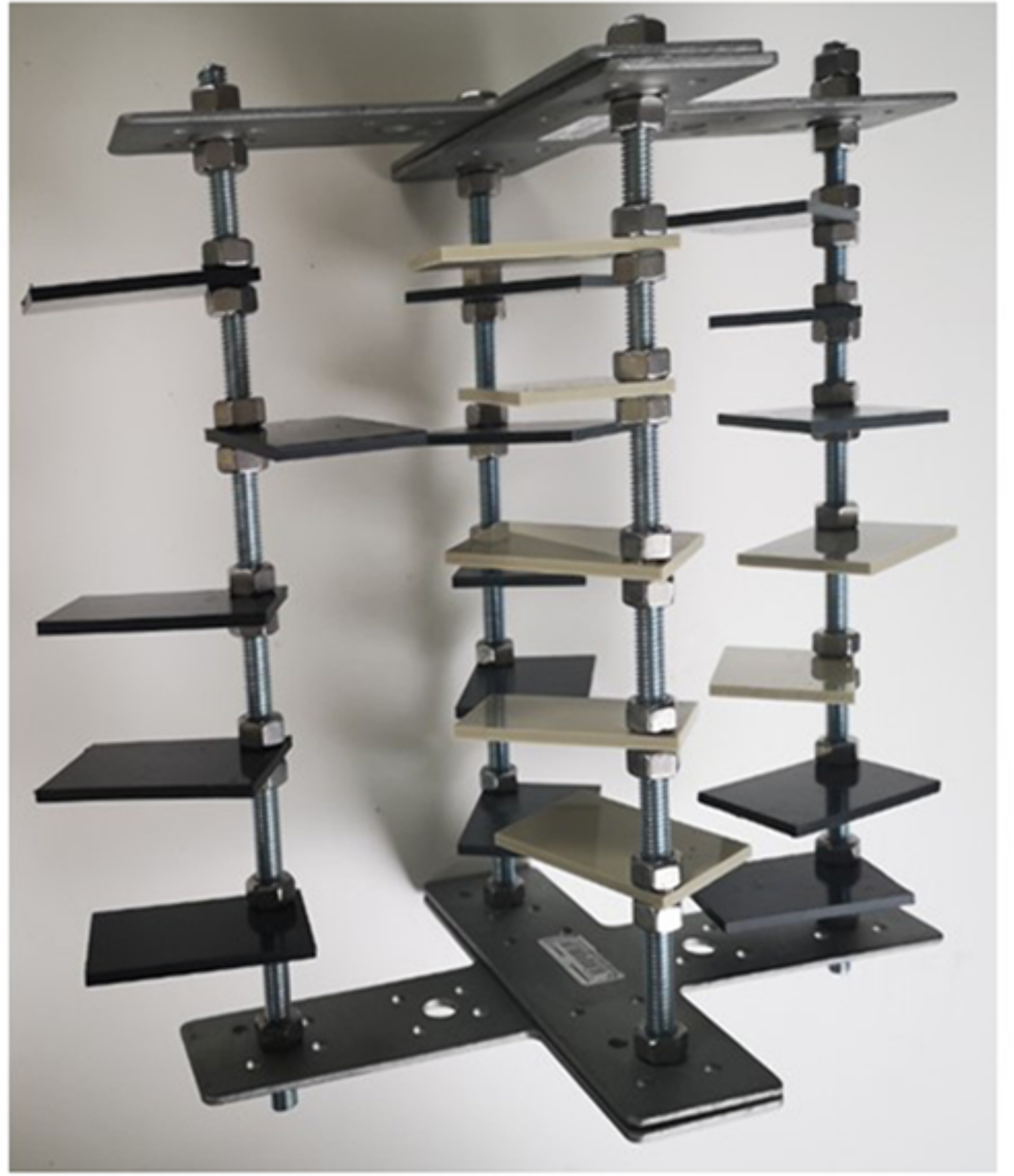

Supplement: S1 Fig — The custom-made device was used to collect plastispheres from the river. The plastic pieces were mounted onto a costum-made device before being inserted into the river. To ensure the device was positioned vertically in the water column, a floating buoy and a weight were attached to the top and the bottom of the device, respectively. (TIF) [file pone.0330754.s005.tif]

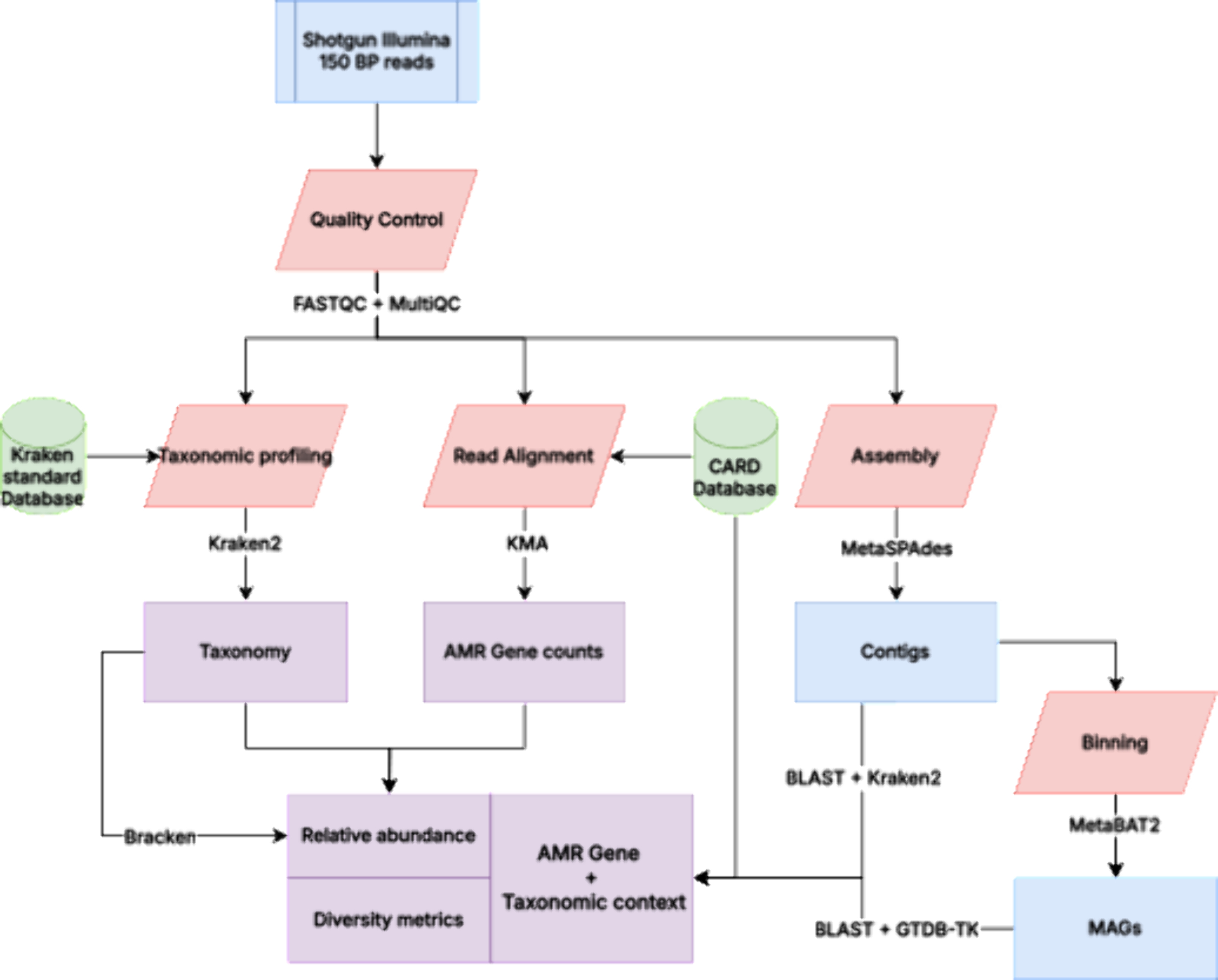

Supplement: S2 Fig — Flow chart of bioinformatics pipeline. (TIF) [file pone.0330754.s006.tif]

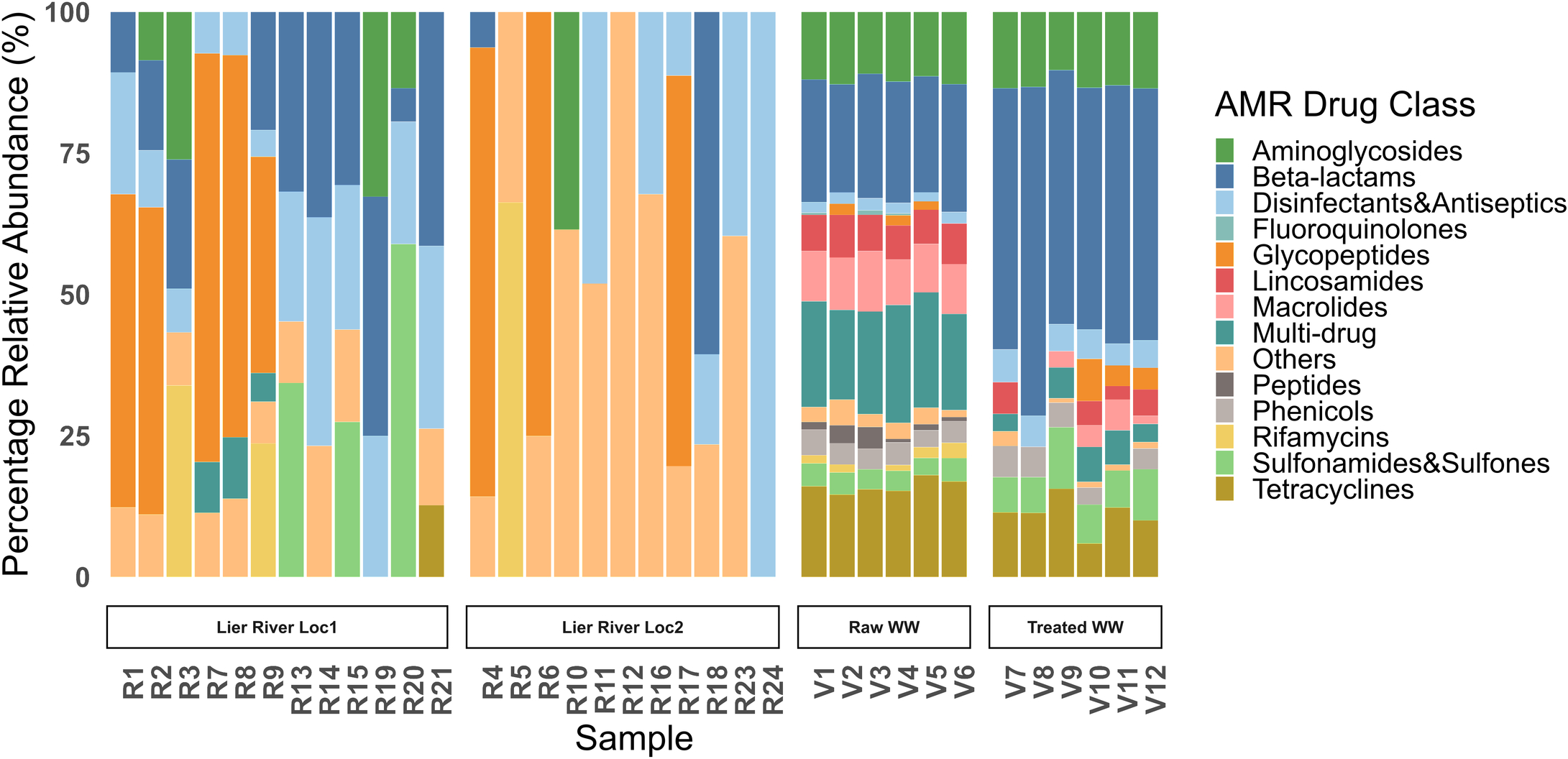

Supplement: S3 Fig — A barplot of the abundance of antimicrobial resistance drug classes in the resistome from the different environments. The length of the bars represents the percentage relative abundance of the drug class, and each color represents one drug class. (TIF) [file pone.0330754.s007.tif]

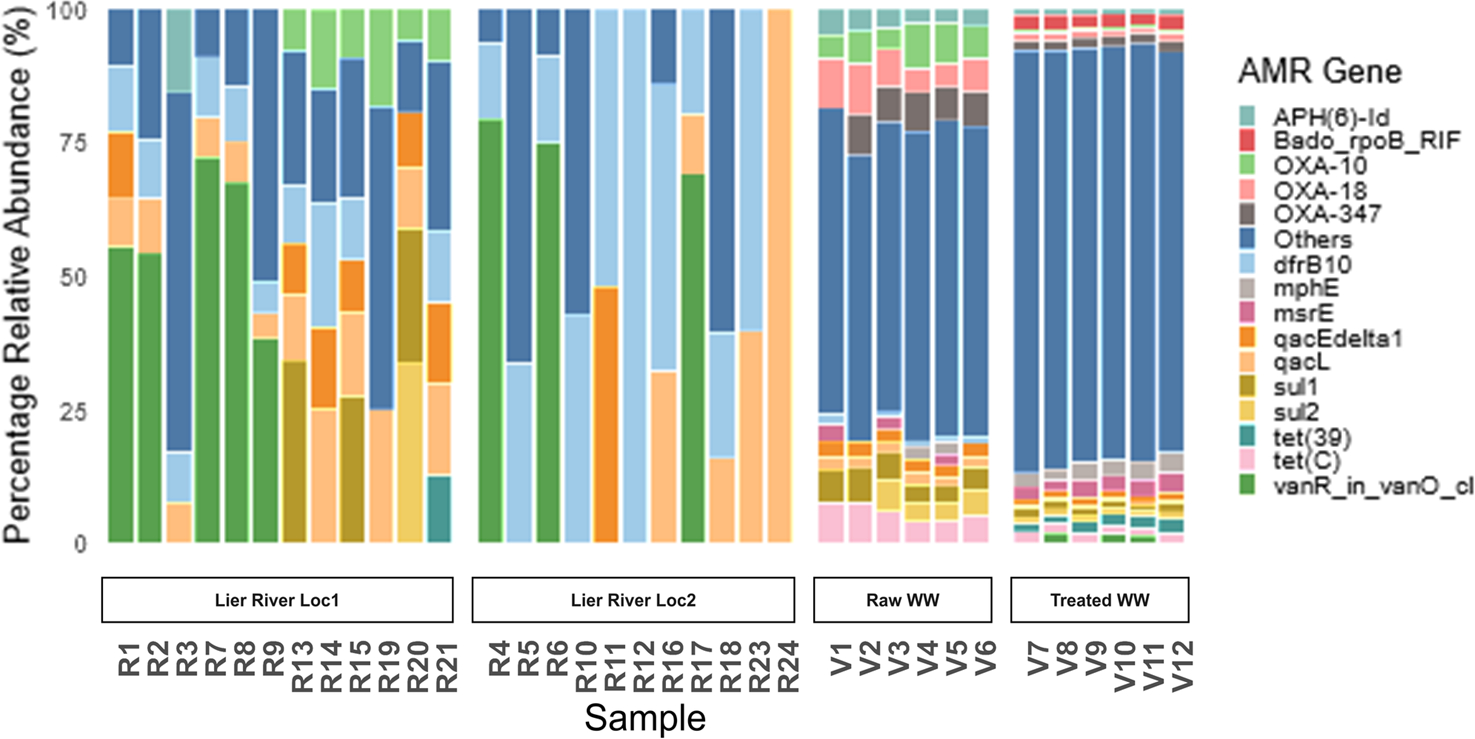

Supplement: S4 Fig — The abundance of ARG. A barplot of the abundance of antimicrobial resistance genes in the resistome from the different environments. The length of the bars represents the percentage relative abundance of the genes, and each color represents one gene. (TIF) [file pone.0330754.s008.tif]
